# *Toxoplasma gondii* bradyzoites and tachyzoites isolation from vitreous of atypical necrotizing retinitis

**DOI:** 10.1186/s12348-018-0151-x

**Published:** 2018-06-15

**Authors:** Ranju Kharel(Sitaula), Sagun Narayan Joshi, Ranjit Sah, Sushila Khadka, Anadi Khatri(KC), Bharat Mani Pokharel

**Affiliations:** 1 B.P. Koirala Lions Centre for Ophthalmic Studies, Maharajgunj Medical Campus, Department of Ophthalmology, Institute of Medicine, Tribhuvan University, Maharajgunj, Kathmandu, Nepal; 2 Vitreo-Retinal Pathology and Surgery, Lumbini Eye Institute, Lumbini, Nepal; 3 Department of Microbiology, Institute of Medicine, Tribhuvan University, Kathmandu, Nepal

## Abstract

**Background:**

Detection of Toxoplasma gondii cysts in vitreous of immunocompetent patient with necrotizing retinitis is extremely rare. We herein report the isolation of Toxoplasma bradyzoites and tachyzoites from the vitreous of healthy person.

**Results:**

A 19-year-old immunocompetent female presented with sudden loss of vision in left eye since 1 week. The BCVA was 6/6 and HM in right and left eye. The left eye finding was suggestive of diffuse necrotizing retinitis with retinal detachment. The IgM and IgG for TORCH infection were negative and HIV, HCV and HBsAg tests were also non reactive. The patient underwent diagnostic and therapeutic vitrectomy with silicon oil installation.

The vitreous toxoplasma IgG titre was found to be significantly raised to 1:16. Bradyzoites of toxoplasma were identified in H&E staining and tachyzoites of Toxoplasma were identified in Giemsa staining of vitreous sample. She received oral clindamycin and oral corticosteroid but the vision could not be restored in left eye.

**Conclusion:**

Hence, atypical toxoplasmosis with necrotizing retinitis is a fulminant condition with the diagnostic and therapeutic challenge.

## Introduction

*Toxoplasma gondii* (*T. gondii*) infects up to a third of the world’s population [1]. This prevalence can be much higher in the areas where hygiene is poor, or raw meat is routinely ingested. Around 1–3% of the infected individuals may develop potentially blinding inflammatory eye disease [2].

Toxoplasmosis is one of the most common global zoonoses and can infect any part of the body. They have a special affinity to the eye—specifically to the tissues of the nervous system including the retina. Since *Toxoplasma* is an intracellular parasite, the retina can sustain the primary insult and can manifest as necrotizing retinitis [2].

The diagnosis is usually based on the clinical appearance of the fundus lesion. Serological tests, imaging techniques, polymerase chain reaction (PCR), histological demonstration of the parasite, or isolation of the organism usually aid in reaching the definitive diagnosis.

However, it must be noted that the clinical diagnosis of ocular toxoplasmosis is supported by laboratory tests only in 60–85% of cases. This is mainly because the sensitivity and specificity of these tests depend on the time of sampling [3].

Demonstration of *T. gondii* cysts within the necrotic retina via biopsy is confirmatory but is rare and extremely difficult to perform [4, 5]. The tachyzoites are known to be capable of independent migration across human vascular endothelium [6]. This could be one of the reasons why very few reports are available on demonstration of *Toxoplasma* organisms in tissue biopsies/cytology [7, 8], necropsy material [9], or enucleated eyes [10, 11]. We hereby, report the confirmation of ocular toxoplasmosis by the isolation of *Toxoplasma* tachyzoites and bradyzoites from the vitreous of an immunocompetent person suspected with unilateral necrotizing retinitis. Written consent from the patient and ethical approval from the Institutional Review Board were obtained.

## Findings

A 19-year-old immunocompetent female presented with painful diminution of vision in the left eye since 10 days. It had a sudden onset with rapid progression. It was not associated with floaters or visual phenomena during the initiation or course of the symptoms. She denies any history of trauma, allergy, or systemic associations except for the history of jaundice 1 month back from which she has now made a total recovery. She is a waitress by profession, non-vegetarian, and gives the history of frequent consumption of undercooked meat of goat, pork, and buffalo.

**Fig. 1:**
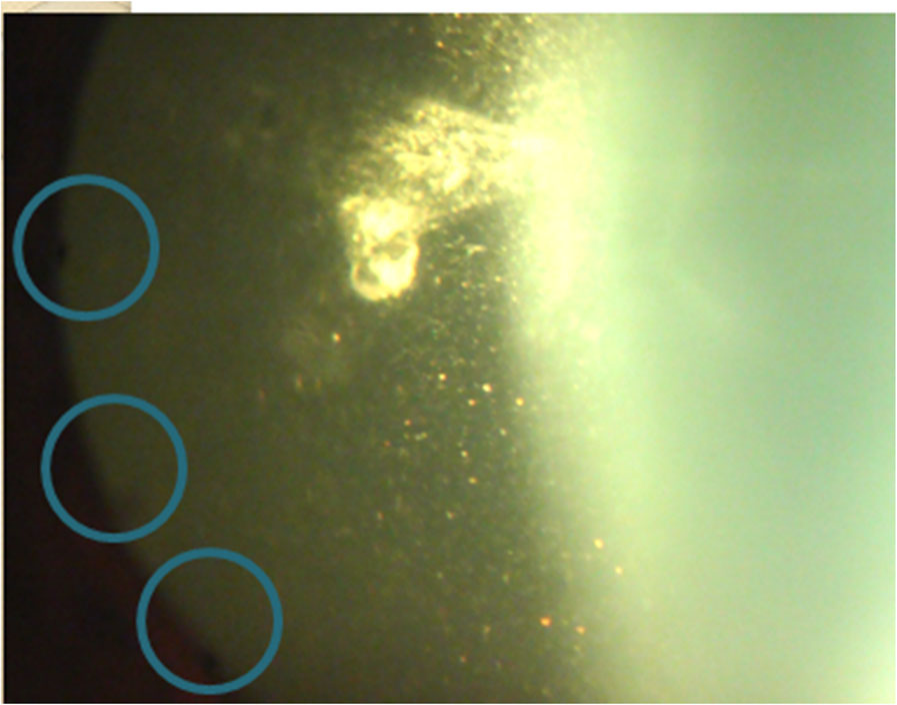
Mutton-fat KPs (brown outline) and Koeppes’ nodules (blue outline)

On examination, vision in the right eye (RE) was 6/6 and in the left eye (LE) was perception of light (PL) with accurate projections. The right eye had grossly normal anterior and posterior segment findings. On the examination of the left eye, there was the presence of circumcorneal congestion; mutton-fat keratic precipitates over the endothelium and Koeppe’s nodules at the pupillary margin of the iris (Fig. 1). The reaction in the anterior chamber was intense with cells 4+ and flare 4+.

**Fig. 2:**
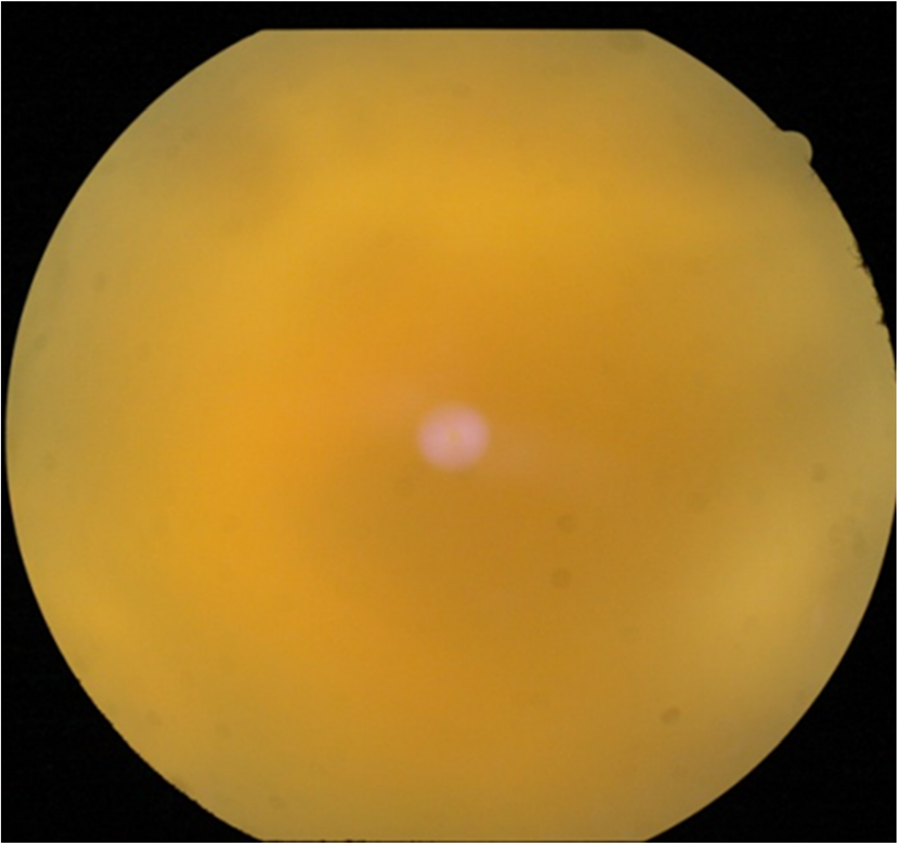
Poor fundal glow in the left eye

On the examination of the posterior segment, the eye had 4+ vitreous haze and poor red reflex. Further details of the fundus could not be appreciated (Fig. 2).

**Fig. 3:**
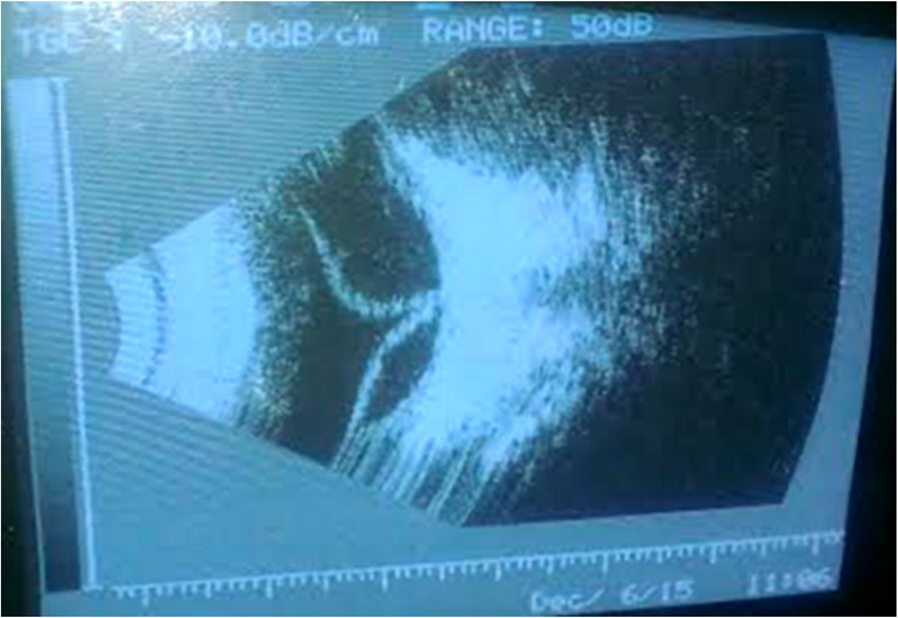
Band-like hyperechogenic structure extending from the vitreous base towards the posterior hyaloid surface and optic nerve head

Intraocular pressure was 15 mmHg in the RE and 13 mmHg in the LE. The ultrasonography of the left eye was mandated immediately. It revealed a band-like hyperechogenic structure extending from the vitreous base towards the posterior hyaloid surface converging around optic nerve head. This radiological finding was persisting in low gain (Fig. 3) which suggested of vitritis along with wide anterior and narrow posterior retinal detachment of funnel shape.

A provisional diagnosis of acute granulomatous panuveitis with a retinal detachment of the left eye was made with acute retinal necrosis (ARN) as a probable cause. Tailored laboratory investigations were sent, and treatment with oral acyclovir and oral corticosteroid was started immediately. The laboratory reports for IgM and IgG for TORCH infection were negative. Serological tests for HIV, HCV, and HBsAg were also negative. PCR for herpes virus family could not be performed due to unavailability. The patient then underwent diagnostic and therapeutic vitrectomy using 23G (25 mm) (0.6 mm bore) cutter via the pars plana route. There was the presence of dense vitreous exudate with multiple atrophic holes near the ora with near total retinal detachment. The optic nerve was found to be slightly pale. The vitreous sample (0.5 ml) was retrieved and sent for cytology and toxoplasma IgG titer.

**Fig. 4:**
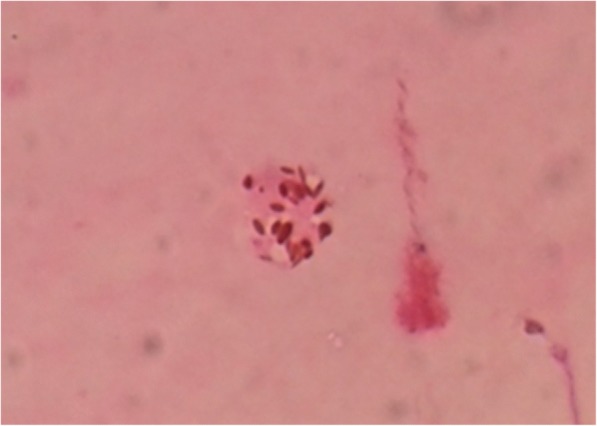
Bradyzoites of *Toxoplasma* were seen in H&E staining

**Fig. 5:**
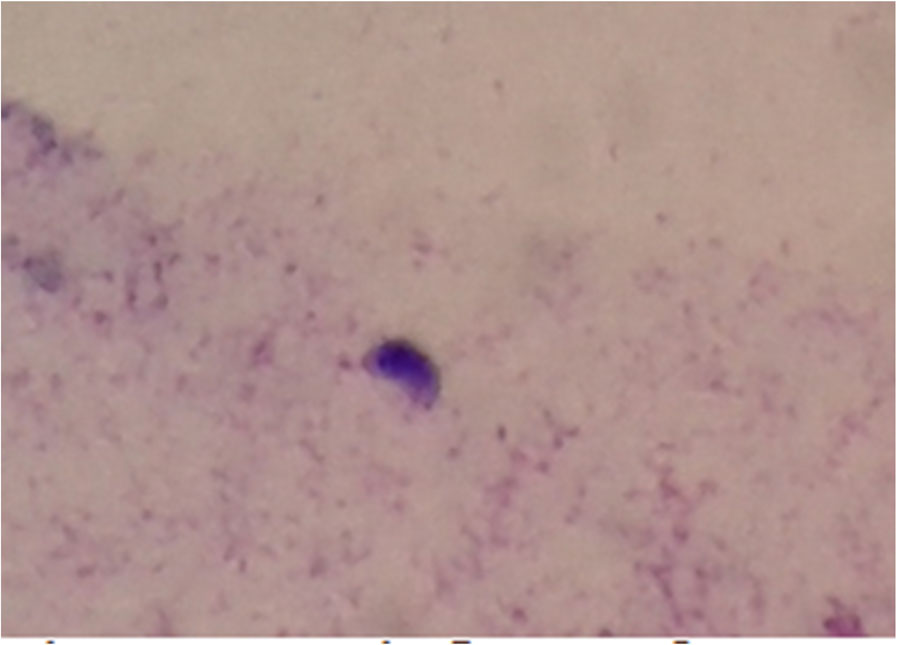
Tachyzoites of *Toxoplasma* were seen in Giemsa staining

Toxoplasma IgG vitreous titer was raised significantly to 1:16. To everyone’s surprise, the microbiological examination of the vitreous yielded bradyzoites of *Toxoplasma* in H&E staining (Fig. 4) and tachyzoites in Giemsa staining (Fig. 5). This confirmed the diagnosis of atypical ocular toxoplasmosis.

**Fig. 6:**
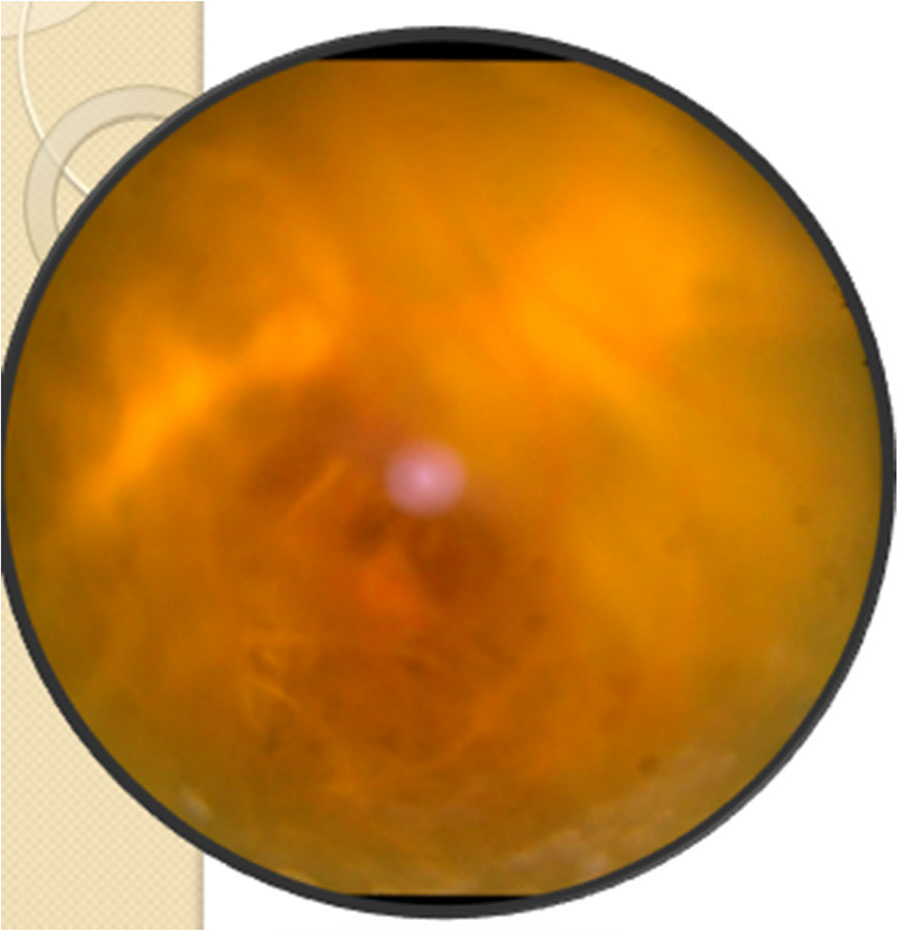
Poor fundal visualization even after vitrectomy

The treatment plan was revised and the patient was started with oral clindamycin and oral corticosteroid, but the visual loss was not permanent as the anatomical integrity of the retina had succumbed to the damage. A pars plana approach to save the retina from further damage was attempted, and silicon oil instillation was tried—but in vain (Fig. 6).

## Discussion

Necrotizing retinopathies represent a diagnostic and therapeutic challenge for all ophthalmologists. Commonest suspected cause is acute retinal necrosis (ARN) which carries a poor visual outcome and a high rate of complications, such as rhegmatogenous retinal detachment [12]. However, as noted in our case, other infectious and non-infectious causes like toxoplasma retinitis and other great mimickers such as syphilis, Behcet’s disease, intraocular lymphoma, and aspergillosis also have to be borne in mind. Inadvertent use of antiviral agents and corticosteroids can be prevented by exploring the causes of necrotizing retinitis besides ARN.

Parasite of *T.* gondii is rarely identified in aqueous/vitreous humor samples from patients with active ocular toxoplasmosis mainly because the parasite proliferation occurs only during the early phase of infection, but the retinal damages are probably caused by subsequent inflammatory responses [13].

Due to the atypical clinical presentations, positive history of intake of undercooked meat, poor response to therapy, and a high clinical suspicion of atypical ocular toxoplasmosis in an immunocompetent person resorted us to proceed to vitreous cytological analysis. The seroprevalence in some part of the world including Nepal can be as high as 50% due to ingestion of water or vegetables contaminated with *T. gondii* oocysts and ingestion of undercooked/raw meat—which are a common dish in some Nepalese community [14].

Though the presence of any titer of IgG against *Toxoplasma gondii* in aqueous or vitreous sample is highly indicative of ocular toxoplasmosis, the sensitivity and the specificity of intraocular antibody detection have been reported only to be 63 and 89%, respectively [13], but positivity can range up to 95% [15, 16]. However, the drawbacks are due to the limitations of the volume of ocular fluid that can be withdrawn, presence low antibody levels present within the specimen, and risk of complications like cataract and endophthalmitis [13]. While PCR is an easier technique for the detection of *Toxoplasma* DNA, the Goldmann-Witmer coefficient (GWC) detects intraocular antibody production and has shown superior results in diagnosing ocular toxoplasmosis when compared to PCR of aqueous humor [17].

Few reports in the literature have described the isolation of *T. gondii* from the eye following an episode of active retinal disease [18, 19]. Usually, it is the proliferative tachyzoite stage where the viable parasites could be isolated. We could isolate both the tachyzoite and bradyzoites form of *T. gondii* in the active stage. This could be due to the rapid transitional stage of tachyzoite to bradyzoites, with the formation of tissue cysts [20].

The help and meticulousness of cytopathologist and microbiologist are equally important to achieve a quick and accurate diagnosis as in our situation. With the advancement in sampling techniques, it may one day be possible to demonstrate this illuding protozoan in a greater proportion of ocular samples sent to the cytology laboratory.

## Conclusion

Demonstration of cyst of *T. gondii* in an ocular fluid sample is extremely difficult with strong suspicion and meticulous search—a timely confirmation of the disease may prevent permanent vision loss.
